# Supplementary material for: Resolving the individual contribution of key microbial populations to enhanced biological phosphorus removal with Raman–FISH
Source: ISME J. 2019 Mar 20;13(8):1933–46. doi: 10.1038/s41396-019-0399-7 (PMC6776032; doi:10.1038/s41396-019-0399-7)
Supplement: Supplementary file 1 — supplementary material [file 41396_2019_399_MOESM1_ESM.pdf]

Supplementary material

Resolving the individual contribution of key microbial populations to enhanced biological phosphorus removal with Raman-FISH

Eustace Y. Fernando[1], Simon Jon McIlroy[1], Marta Nierychlo[1], Florian Herbst-Alexander[1], Francesca Petriglieri[1], Markus C. Schmid[2], Michael Wagner[2], Jeppe Lund Nielsen[1], and Per Halkjær Nielsen[1]

[1] Center for Microbial Communities, Department of Chemistry and Bioscience, Aalborg University, Aalborg, Denmark.

[2] University of Vienna, Department of Microbial Ecology and Ecosystem Science, Research Network "Chemistry meets Microbiology", Vienna, Austria.

Figure S1: Raman reference spectra.

Figure S2: Comparison of Raman spectra.

Figure S3: Linear relationship between the poly-P material density and average Raman intensity.

Figure S4: Raman heatmap image of a dried poly-P droplet.

Figure S5: Material density-based Raman calibration plots (PHA and glycogen).

Figure S6: Fluorophore Bleaching experiment.

Figure S7: Comparison of Raman spectra of the *T. elongata.*

Figure S8: Loss of storage polymers (poly-P and PHA) due to fixation and FISH.

Figure S9: Dynamics of intracellular poly-P in *Tetrasphaera* and poly-P, PHA, and glycogen in *Ca.* Accumulibacter in the different treatment stages of 8 EBPR plants.

Figure S10: The fraction of P being taken up in *T. elongata* cells during pure culture P uptake/release experiments

Figure S11: Comparison of qFISH biovolume fractions (% of EUBmix) of PAOmix, PAO651, and Prop207 in *in situ* samples of all the WWTPs

Table S1: WWTP size (population equivalents), influent wastewater type, and influent/effluent ortho-P concentrations.

Table S2: Bulk ortho-P concentrations in different process tanks.

Supplementary text

1. Sampling area in the lateral dimension
2. Validation of the Raman estimation of poly-P with chemical analysis of the bulk medium orthophosphate levels
3. Quantification of *Ca*. Accumulibacter by FISH
4. Example calculation of per-cell poly-P quantification in *T. elongata* P-uptake/release
5. The detection threshold for a given analyte
6. Example of P mass balance calculations

Supplementary references

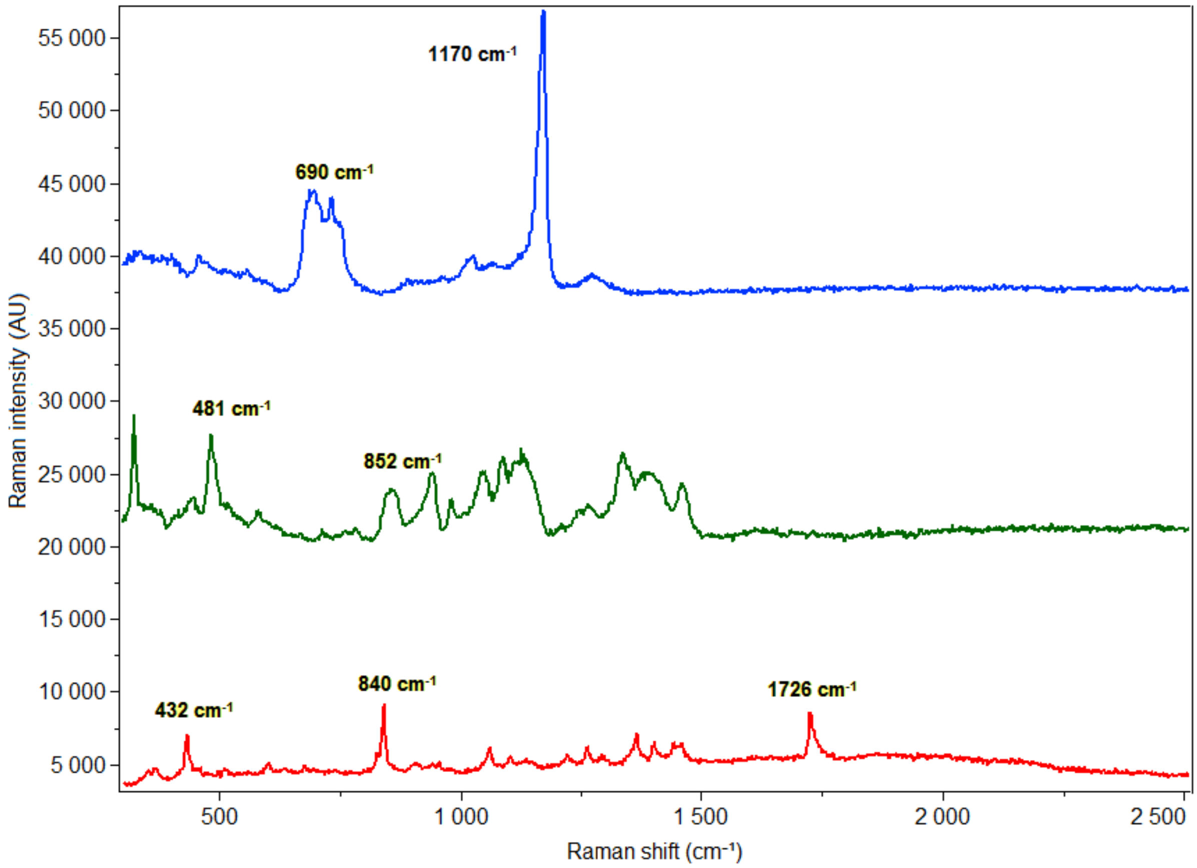

Figure S1: Raman reference spectra of sodium poly-P (blue), glycogen (green), and poly-3-hydroxybutyrate-co-hydroxyvalarate (red) showing marker bands at 1170 cm^{-1} (PO_2^- stretching vibrations), 481 cm^{-1} (C-C skeletal deformation), and 1726 cm^{-1} (CC skeletal stretch), respectively. The chemical standard for poly-P used here is sodium hexametaphosphate. Since the Raman analysis for poly-P relies on the v(-P-O-P-) stretching vibrations occurring at the 1170 cm^{-1} wavenumber region, the degree of polymerization in poly-P does not change the Raman return signal for the v(-P-O-P-) bond system, which is always between the 1160 – 1170 cm^{-1} wavenumber region[1,2,3]. Glucose has characteristic peaks at 911 cm^{-1}, 1060 cm^{-1}, and 1125 cm^{-1}, and therefore, will not interfere with Raman peak assignments of glycogen[4].

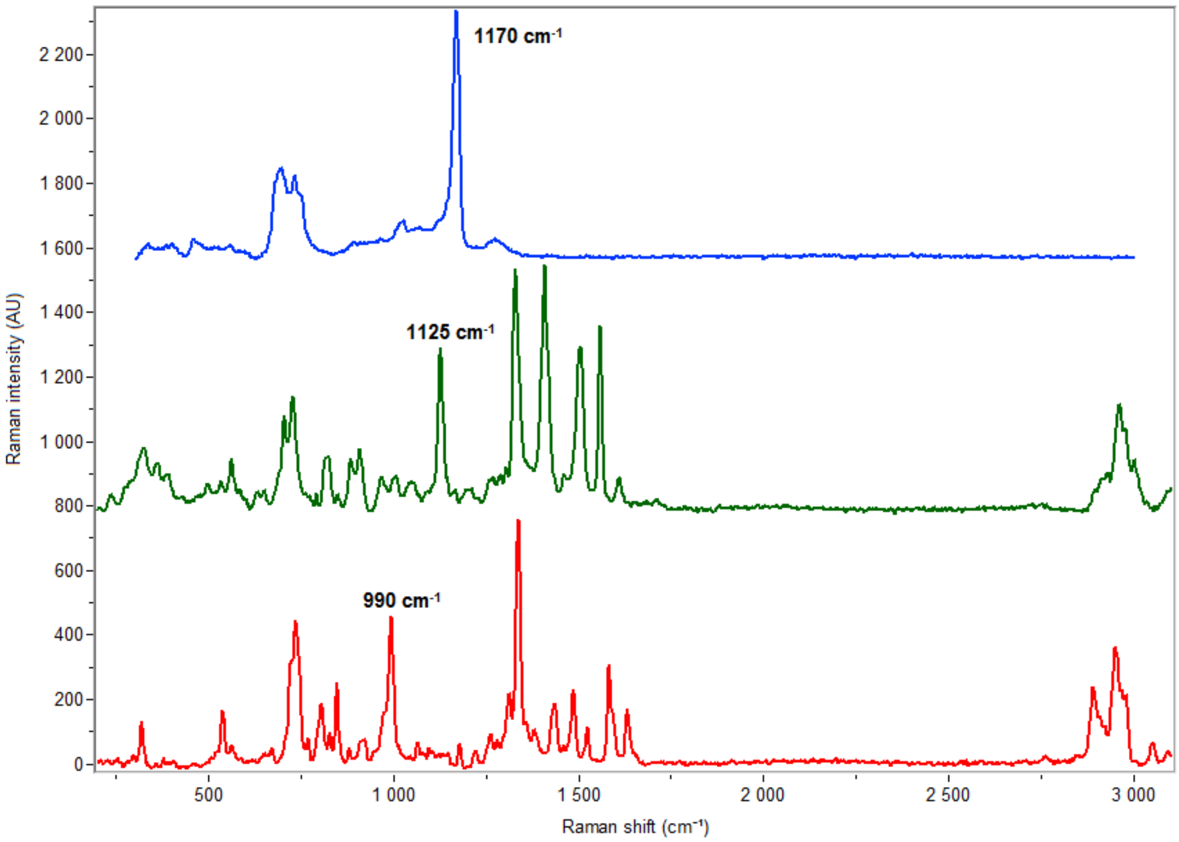

Figure S2: Comparison of Raman spectra of poly-P (blue), ATP (green), and AMP (red). The Raman marker peak at 1170 cm^{-1} used for poly-P is not observed in spectra of ATP (1125 cm^{-1}) and AMP (990 cm^{-1}).

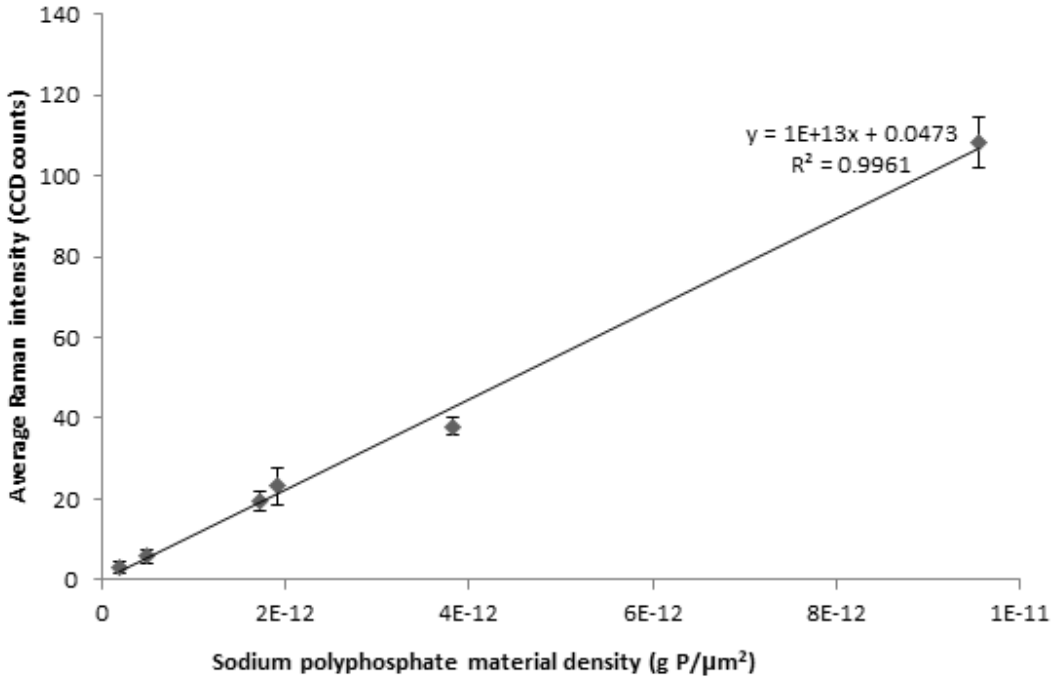

Figure S3: Linear relationship between the poly-P material density and average Raman intensity of the poly-P marker band (at 1170 cm^{-1}). Error bars are standard deviation in average Raman counts of triplicate polyphosphate droplets at each material density.

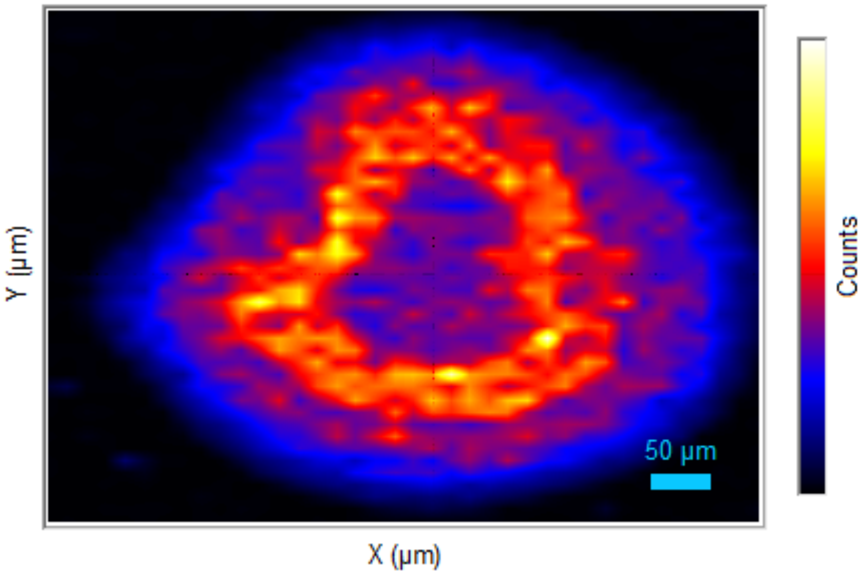

Figure S4: Raman heatmap image of a dried poly-P droplet used for the determination of the calibration coefficient (k).

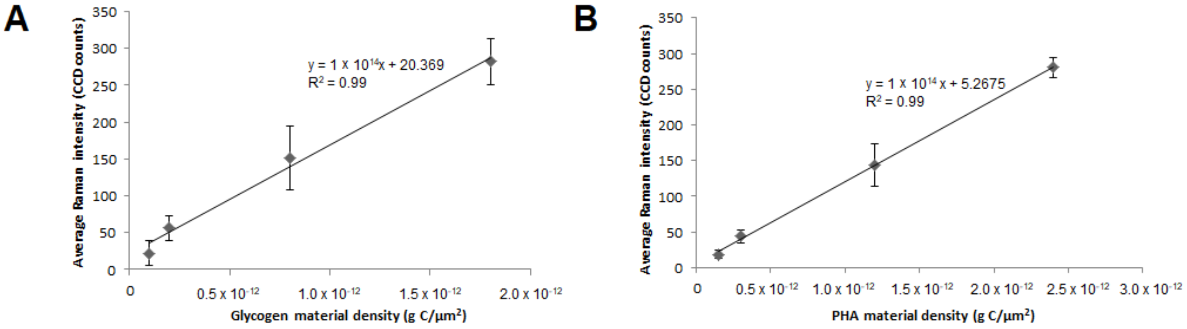

Figure S5: Material density-based Raman calibration plots for (A) glycogen and (B) PHA. Error bars are standard deviation in average Raman counts of triplicate PHA/glycogen droplets at each material density.

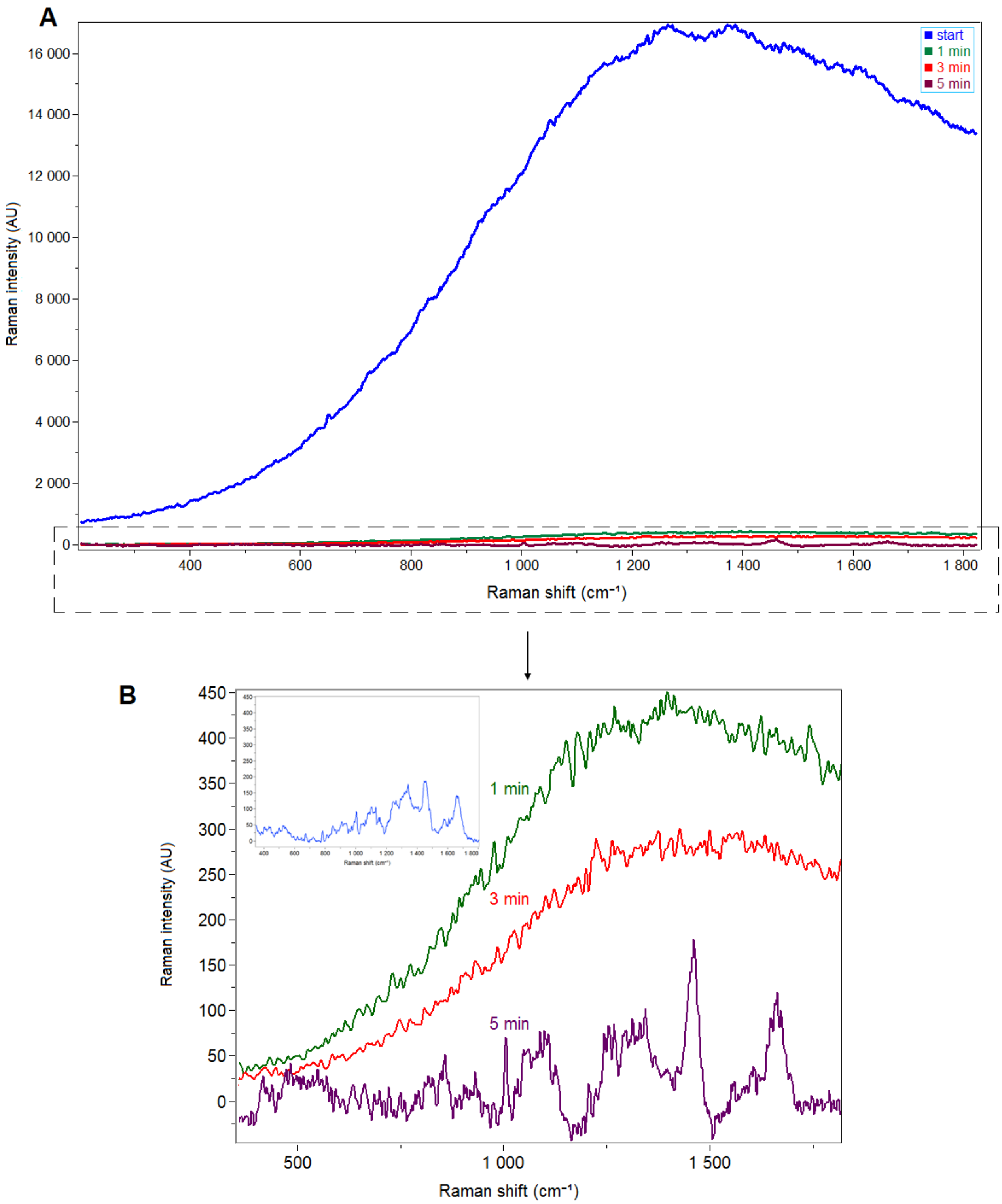

Figure S6: A) Cy3 bleaching experiment with *T. elongata* cells hybridized with the EUBmix labeled with Cy3. Panel A shows Raman spectra recorded after different times of bleaching. Panel B displays a re-scaled (1-5 min) set of spectra. After 5 min of bleaching, the Raman spectrum of *T. elongata* was unaffected by Cy3 fluorescence. Panel B inset shows a Raman spectrum of *T. elongata* cells hybridized with the EUB338 labeled with FLUOS, indicating that the FLUOS fluorophore does not interfere with Raman measurements.

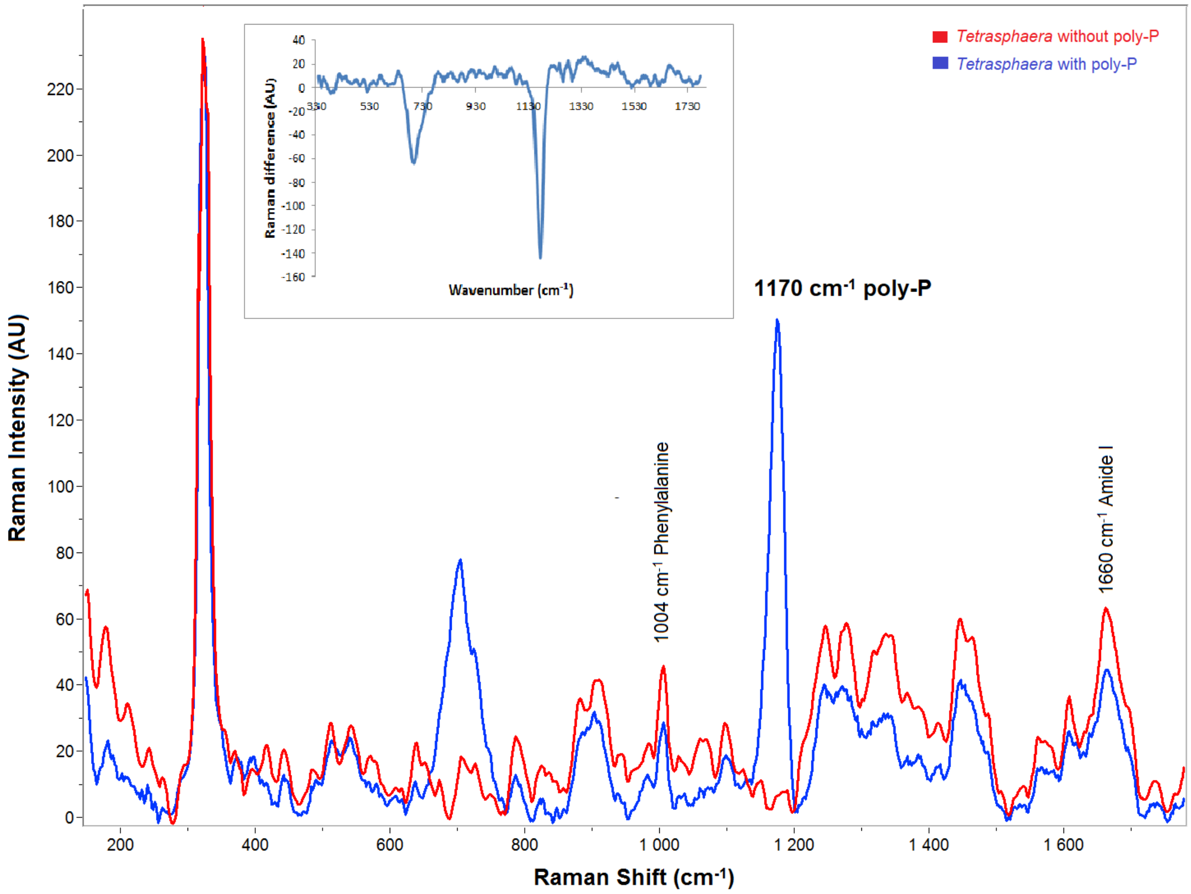

Figure S7: Comparison of Raman spectra of the *T. elongata* pure culture during P-release and P-uptake conditions. Inset – Raman difference spectrum between *Tetrasphaera* cells without poly-P (red) and *Tetrasphaera* cells with poly-P (blue). Missing signature peaks for glycogen and PHA demonstrate that these storage compounds were not produced in significant amounts during the experiment.

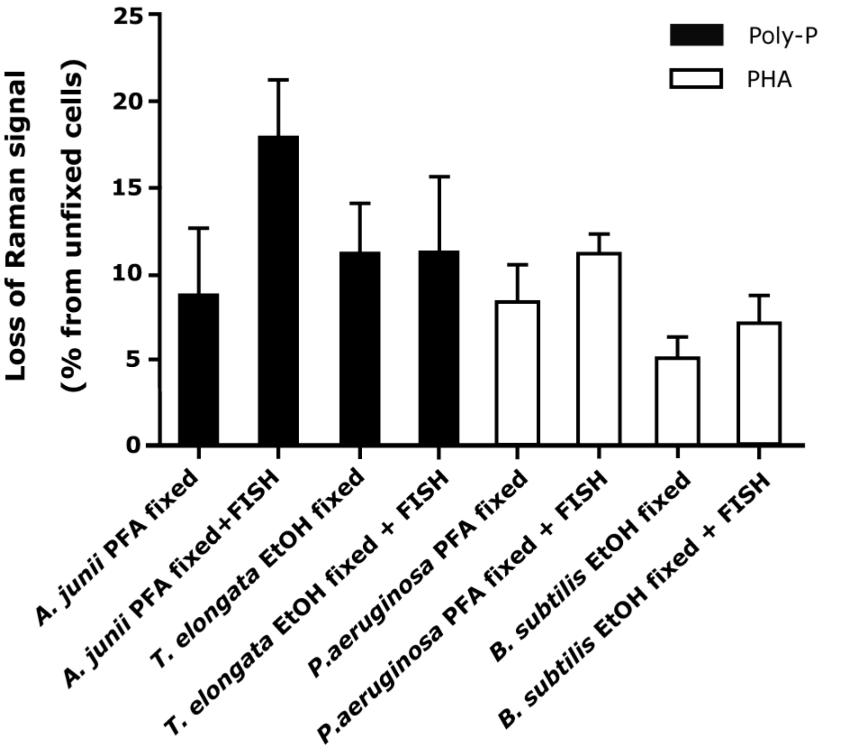

Figure S8: Loss of storage polymers (poly-P and PHA) due to fixation and FISH in the Gram-positive strains *T. elongata* and *B. subtilis* as well as in the Gram-negative strains *A. junii* and *P. aeruginosa.* All fixed cells were stored at 4°C for eight weeks (mean ± SD in error bars, n = 100 individual random cells in each instance).

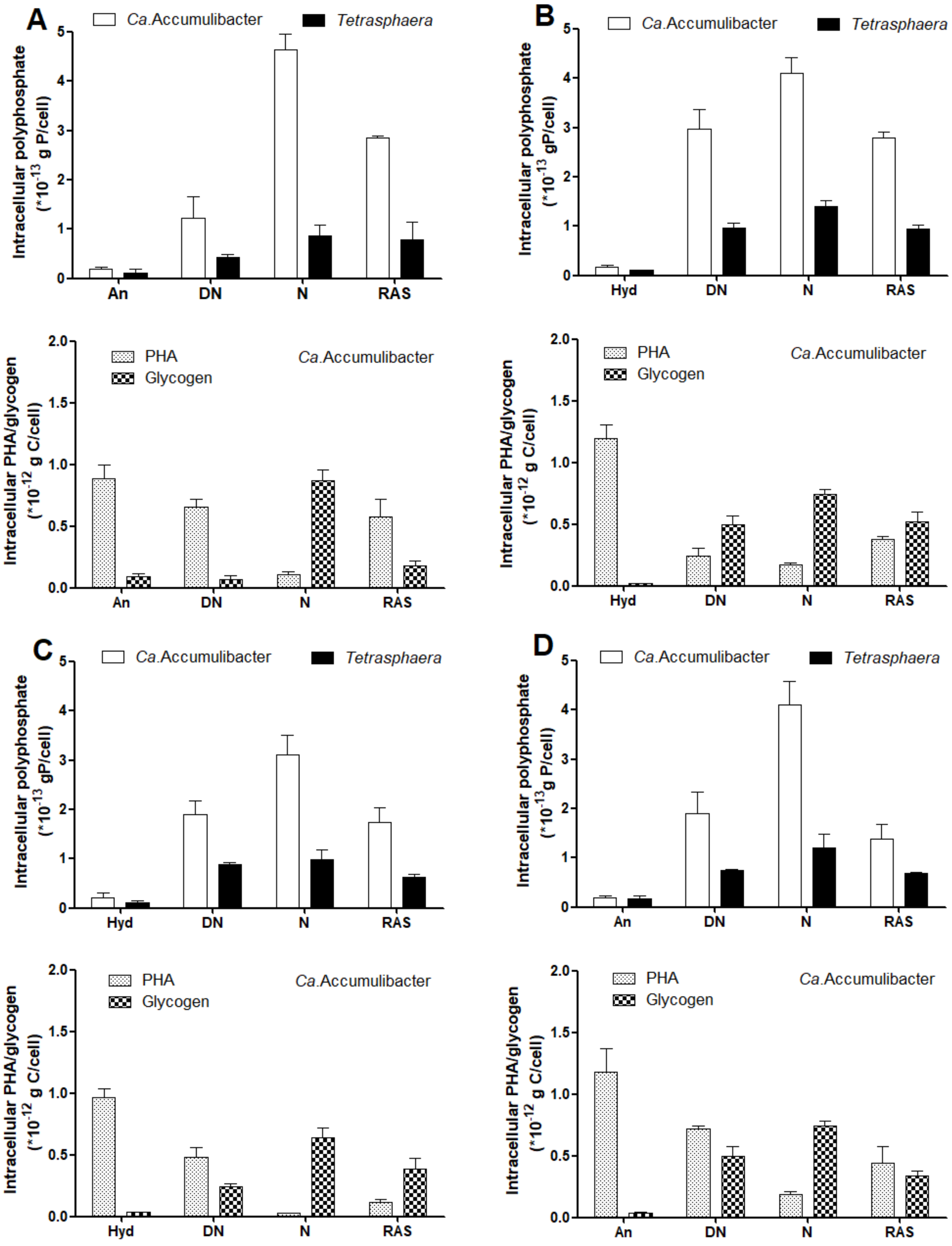
A
B
C
D
Ca.Accumulibacter
Tetrasphaera
PHA
Glycogen
Intracellular polyphosphate (*10^-13 g P/cell)
Intracellular polyphosphate (*10^-13 gP/cell)
Intracellular PHA/glycogen (*10^-12 g C/cell)
An
Hyd
DN
N
RAS

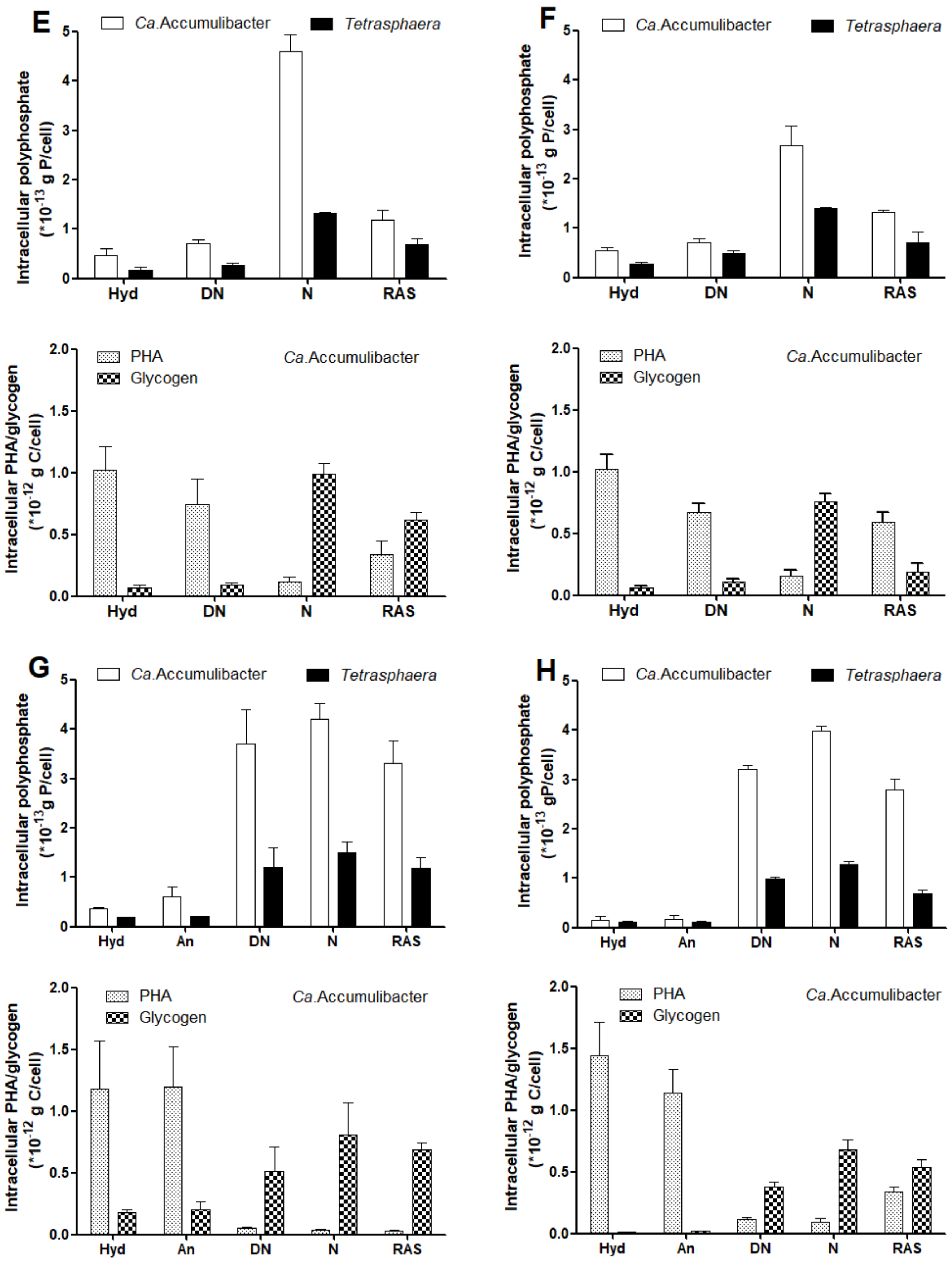
E
F
G
H
Ca.Accumulibacter
Tetrasphaera
PHA
Glycogen
Intracellular polyphosphate (*10^-13 g P/cell)
Intracellular PHA/glycogen (*10^-12 g C/cell)
Hyd
An
DN
N
RAS

Figure S9: Dynamics of intracellular poly-P in *Tetrasphaera* and poly-P, PHA, and glycogen in *Ca.* Accumulibacter in the different treatment stages of 8 EBPR plants: (A) Hjoerring, (B) Randers, (C) Viby, (D) Bjerringbro, (E) Aalborg East, (F) Aalborg West, (G) Egaa, and (H) Aaby. It should be noted that the poly-P content in both PAOs in all plants was lower in the return sludge from the settler (RAS stream) than in the aerobic N tanks. Similarily, in the settler, the intracellular glycogen content in *Ca.* Accumulibacter was also slightly reduced, while PHA levels increased (see also Fig. 4). This pattern indicates the presence of anaerobic conditions in the settler together with the availability of organic carbon. Alternatively, glycogen/poly-P were used for maintenance in the settler(7). The residence time for the biomass in the settlers is 10-15 h, making it possible that anaerobic conditions may have developed. Anaerobic conditions in the clarifiers are not intended as released ortho-P will increase the P-effluent concentration (mean ± SD in error bars, n = 100 individual random cells in each instance).

Table S1: WWTP size and configuration, industrial wastewater contribution as well as influent/effluent P concentrations (average values from the sludge sampling month).

WWTP location	Plant size (population equivalents)	Plant configuration	Wastewater type and (%) industrial COD contribution	Influent P concentration (mg P L^{-1})	Effluent P concentration (mg P L^{-1})
Hjoerring	160 000	Recirculation	30	5.2	0.2
Randers	77 000	Recirculation	5	5.9	0.3
Viby	100 000	Recirculation	6	3.3	0.3
Bjerringbro	30 000	Alternating	20	2.5	0.03
Aalborg East	110 000	Alternating	10	9.7	0.6
Aalborg West	330 000	Alternating	30	6.3	0.5
Egaa	112 000	Alternating	10	3.6	0.2
Aaby	107 000	Alternating	30	5.7	0.5

Table S2: Bulk orthophosphate concentrations at the time of sampling at different process tanks of all WWTPs investigated (mean ± SD).

WWTP location	Ortho-P concentration (mg P L^{-1})				
	Hydrolysis tank	Anaerobic tank	Denitrification tank	Nitrification tank	Return activated sludge
Hjoerring	-	2.30 ± 0.40	2.52 ± 0.22	0.42 ± 0.03	0.82 ± 0.09
Randers	-	21.7 ± 1.31	1.42 ± 0.62	0.45 ± 0.06	5.30 ± 0.75
Viby	18.8 ± 2.1	-	0.94 ± 0.03	0.57 ± 0.08	4.13 ± 0.69
Bjerringbro	-	10.7 ± 1.03	2.06 ± 0.44	0.55 ± 0.10	1.22 ± 0.20
Aalborg East	88.8 ± 3.8	-	3.27 ± 0.64	0.53 ± 0.06	8.30 ± 1.60
Aalborg West	114 ± 2.7	-	7.07 ± 0.92	1.01 ± 0.07	2.50 ± 0.40
Egaa	24.9 ± 1.4	15.2 ± 0.48	0.79 ± 0.08	0.28 ± 0.10	0.85 ± 0.05
Aaby	76.9 ± 2.1	21.5 ± 0.87	0.60 ± 0.05	0.33 ± 0.03	1.47 ± 0.22

Supplementary text

1. Sampling area in the lateral dimension

The theoretically calculated laser illuminated diameter is given by;

1.22λ/NA, where λ and NA are the laser wavelength and the numerical aperture of the microscope objective respectively. However, the actual illuminated diameter can vary up to 300% of the theoretically calculated value due to imperfections in the optical components and the finite bandwidth of the laser[(8)]. The actual laser spot diameter can be empirically approximated using the 10/90 criterion method as detailed in Zoubir, (2012)[(8)] and was found to be 2.6 ± 0.18 µm.

The sampling depth with the settings used can be approximated as detailed in Zoubir, (2012)[(7)] and was approximately 3.8 µm.

2. Validation of the Raman estimation of poly-P with chemical analysis of the bulk medium orthophosphate levels

From DAPI stained cell counts, the estimated *T. elongata* cell number was $7.9 * 10^7 \pm 1.7$ cells/mL

Therefore, in poly-P "full" state (end aerobic phase), the phosphate fraction inside *T. elongata* cells as poly-P, can be estimated using Raman spectroscopy, as;

$1.1 * 10^{-13}$ g cell^{-1} * $7.9 * 10^7$ cells mL^{-1} = **8.7 mg P/L**

The difference P in bulk medium between the start and the end of the aerobic half of the experiment, determined by chemical analysis was **10.7** mg P/L (See figure below).

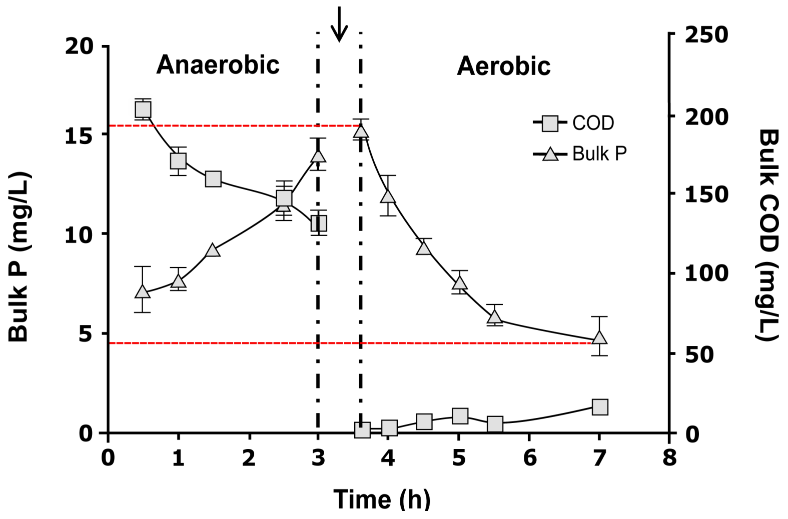

Figure. The fraction of P being taken up in *T. elongata* cells in the aerobic half of the experiment is marked with the two red lines in the figure. That difference, measured by chemical analysis is 10.7 mgP/L.

The Raman estimation of poly-P bound P fraction at the end of the aerobic phase (8.7 mg P/L) and bulk liquid orthophosphate uptake (10.7 mg P/L) during the aerobic stage are approximately the same (there is about a 2 mg/L discrepancy between the two values). Incorporation of some orthophosphate into non-poly-P organic phosphate fraction (i.e., nucleic acids, membrane lipids) during aerobic incubation may account for the 2 mg/L discrepancy seen here, because the Raman method can only account for the P stored as poly-P and not other forms of P.

The sampling volume during the experiment was 1 mL/sample and the vial size was 250 mL. The pH was monitored at the beginning, at the washing stage, and at the end of the P cycling experiment and was never found to deviate away from 7.0-7.2. Sampling was done every 30 min during P uptake/release tests.

3. Quantification of *Ca.* Accumulibacter by FISH

A qFISH experiment was carried out with the use of PAOmix (PAO462, PAO651, and PAO846) probe set and the Prop207 probe (covering most of the *Propionivibrio* spp.)[10] in order to investigate if the use of PAO651 probe alone would lead to substantial underestimation of *Ca*. Accumulibacter in the *in situ* samples (see figure below). qFISH was carried out using the same procedure as described in the materials and methods.

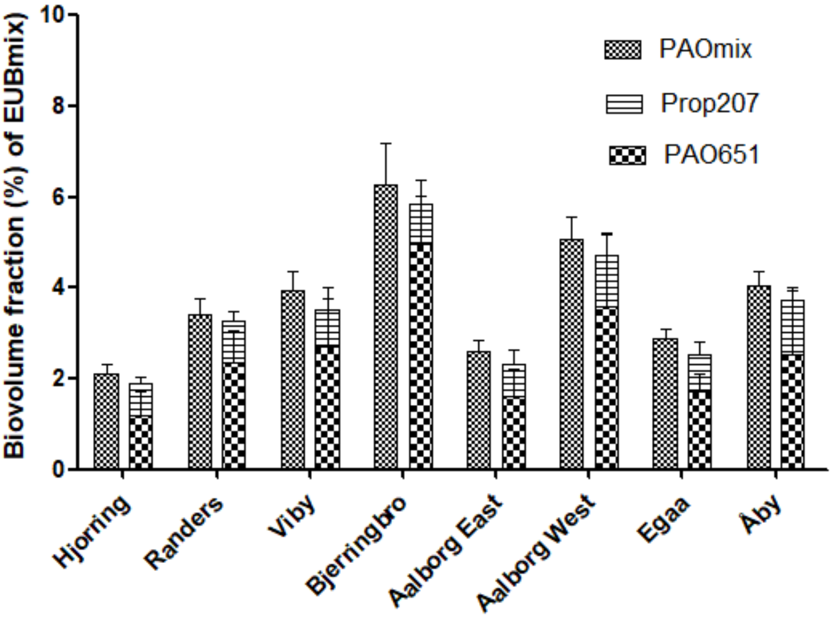

Figure: A comparison of qFISH biovolume fractions (% of EUBmix) of PAOmix, PAO651, and Prop207 in *in situ* samples of all the WWTPs in this study. The cumulative biovolume fractions of PAO651 and Prop207 were always greater than 90% of the biovolume fraction of PAOmix probe set (error bars – mean ± SD).

4. Supplementary calculation-2: Example calculation of per-cell poly-P quantification in *T. elongata* P-uptake/release

T. elongata populations that were analysed in poly-P "full" and "empty" states in the uptake release experiment, under identical instrument settings as described in the materials and methods section, to determine the calibration coefficient value. The average Raman intensities obtained for poly-P "full" and "empty" states were 58.25 CCD counts and 10.05 CCD counts respectively (n = 100 individual *T. elongata* cells).

The average 2D area occupied by a single *T. elongata* cell, when mounted on CaF_2 Raman substrate was estimated to 3.79 ± 0.22 μm^2, using the image processing software, ImageJ (n = 100 cells).

The calibration coefficient (k) for poly-P under these conditions and instrument settings was demonstrated to be $4.98 * 10^{-16}$ g P $\mu m^{-2} counts^{-1}$ (materials and methods section)

In the case of poly-P "full" and "empty" populations, for absolute quantification;

Poly-P per cell = k * average Raman CCD counts * estimated cell area

For *T. elongata* poly-P "full"state;

$4.98 * 10^{-16}$ g P $\mu m^{-2} counts^{-1}$ * 58.25 counts * 3.79 μm^2/cell = $1.1 * 10^{-13}$ g P/cell

For *T. elongata* poly-P "empty" state;

$4.98 * 10^{-16}$ g P $\mu m^{-2} counts^{-1}$ * 10.05 counts * 3.79 μm^2/cell = $0.19 * 10^{-13}$ g P/cell

5. The detection threshold for a given analyte

The limit of detection for a peak- assigned Raman band for an analyte is given by:

$I_{Raman} > 2 * \sqrt{I_{Raman} + I_{Background}}$ [9]

When an analyte was below this threshold, (i.e., glycogen in *Tetrasphaera* cells), it was considered that the analyte was below the limit of detection (LOD).

6. Supplementary calculation – 1: Example of P mass balance calculations

For all plants: Activated sludge (SS; suspended solids): Approx. 10% of organic matter (VSS, volatile suspended solids) is assumed to be cell biomass[5]. All plants investigated were very similar in type of wastewater and design/operation, so same value has been used for all plants.

P is assumed to be 1% of the total biomass[5,6] so assimilated P/g SS:

mg P/g SS = 10 mg P/gSS * (10/100) * (1/0.65) = 1.54 mg P/gSS, where VSS=0.65*SS[6].

The ratio 0.65 varied between 0.62 – 0.67 in the plants investigated and an average value of 0.65 was applied.

Number of cells per g/VSS: $1*10^{12}$ cells/g VSS[5].

Mean biovolume per cell: 2 μm^3/cell[5].

Mean biovolume *Tetrasphaera*: 2.9 μm^3/cell (this study).

Mean biovolume *Ca.* Accumulibacter: 9.2 μm^3/cell (this study).

Poly-P loss from analysed cells during the FISH procedure for both Gram-positive and Gram-negative cells was found to be between 8% - 16%. Therefore, the calculations have been adjusted for a loss of 12% (average).

Hjoerring WWTP

For *Tetrasphaera*:

Biovolume of *Tetrasphaera* in the VSS*:*

Tetrasphaera biovolume = (6.5/100) * 2 μm^3/cell * $1\cdot10^{12}$ cells/g VSS = 1.3 *10^{11} μm^3/gVSS

Poly-P per unit biovolume:

In the aerobic tank: 8.6 *10^{-14} g poly-P /cell

Tetrasphaera cell volume = 2.9 μm^3/cell

g poly-P/μm^3 = 8.6*10^{-14} g P $cell^{-1}$ /2.9 μm^3 $cell^{-1}$ = 3 *10^{-14} g P/μm^3

poly-P in *Tetrasphaera* =

1.3 * 10^{11} μm^3/gVSS * 3 *10^{-14} g P/μm^3 = 3.9 * 10^{-3} g P/g VSS

Therefore, poly-**P per g SS** = 3.9 mg P/g VSS / 0.65 = **6 mg poly-P/g SS.**

To account for the P-loss during FISH procedure; 6 mg poly-P/g SS * 112% = 6.7 mg polyP/g SS

For *Ca.* Accumulibacter

Biovolume of *Ca.* Accumulibacter in the VSS*:*

Ca. Accumulibacter biovolume = (1.2/100) * 2 μm^3/cell * $1\cdot10^{12}$ cells/g VSS = 2.4 *10^{10} μm^3/gVSS

Poly-P per unit biovolume:

In the aerobic tank: 4.6 * 10^{-13} g P /cell

Ca. Accumulibacter cell volume = 9.2 μm^3 /cell

g poly-P/μm^3 = 4.6 * 10^{-13} g P $cell^{-1}$ / 9.2 μm^3 $cell^{-1}$ = 5 *10^{-14} g P/ μm^3

Poly-P in *Ca.* Accumulibacter =

2.4 * 10^{10} μm^3/gVSS * 5 *10^{-14} gP/ μm^3 = 1.2 * 10^{-3} g P/g VSS

Therefore, poly-**P per g SS** = 1.2 mg P/g VSS / 0.65 = **1.86 mg poly-P/g SS**

To account for the P-loss during FISH procedure; 1.85 mg poly-P/g SS * 112% = 2.07 mg poly-P/g SS

Supplementary references

1. Majed N, Matthäus C, Diem M, & Gu AZ. Evaluation of intracellular polyphosphate dynamics in enhanced biological phosphorus removal process using Raman microscopy. *Environ Sci Technol.* **43,** 5436-5442 (2009).

2. Majed N & Gu AZ. Application of Raman microscopy for simultaneous and quantitative evaluation of multiple intracellular polymers dynamics functionally relevant to enhanced biological phosphorus removal processes. *Environ Sci Technol.* **44,** 8601-8608 (2010).

3. Majed N, Chernenko T, Diem M, & Gu AZ. Identification of functionally relevant populations in enhanced biological phosphorus removal processes based on intracellular polymers profiles and insights into the metabolic diversity and heterogeneity. *Environ Sci Technol*. **46,** 5010-5017 (2012).

4. Shao J, Lin M, Li Y, Li X, Liu J, Liang J, Yao H. In vivo blood glucose quantification using Raman spectroscopy. *Plos One*, 7(10): e48127 (2012)

5. Frølund B, Palmgren R, Keiding K, & Nielsen PH. Extraction of extracellular polymers from activated sludge using a cation exchange resin. *Water Res* **30**(8):1749-1758 (1996).

6. Wong M-T, Tan FM, Ng WJ, & Liu W-T. Identification and occurrence of tetrad-forming Alphaproteobacteria in anaerobic–aerobic activated sludge processes. *Microbiology* **150**(11):3741-3748 (2004).

7. Brdjanovic D, et al. Impact of excessive aeration on biological phosphorus removal from wastewater. *Water Res* **32**(1):200-208 (1998).

8. Zoubir, A. (Ed.). Raman imaging: techniques and applications (Vol. 168). Springer: 11-13 (2012).

9. Vandenabeele, P., Jehlička, J., Vítek, P., & Edwards, H. G. M. On the definition of Raman spectroscopic detection limits for the analysis of biomarkers in solid matrices. *Planetary and Space Science*, 62(1), 48-54 (2012).

10. Albertsen M, McIlroy SJ, Stokholm-Bjerregaard M, Karst SM, & Nielsen PH. "*Candidatus* Propionivibrio aalborgensis": a novel glycogen accumulating organism abundant in full-scale enhanced biological phosphorus removal plants. *Front Microbiol.* **7,** 1033 (2016).
